# Effects of a resistance training program on chemotherapy-induced peripheral neuropathy, cancer-related fatigue, muscle strength and physical activity in colorectal cancer patients undergoing chemotherapy: a pilot randomized controlled trial

**DOI:** 10.1007/s12094-026-04371-z

**Published:** 2026-04-30

**Authors:** Carlos Martín-Sánchez, Eduardo José Fernández-Rodríguez, Emilio Fonseca-Sánchez, Yolanda López Mateos, Sofía Espinal-Matos, Javier Martín-Vallejo, Alberto García-Martín, Juan Jesús Cruz-Hernández, Juan Luis Sánchez González

**Affiliations:** 1 Departamento de Enfermería y Fisioterapia, Universidad de Salamanca, Instituto de Investigación Biomédica de Salamanca (IBSAL), Campus Miguel de Unamuno s/n, 37007 Salamanca, Spain; 2 Departamento de Medicina, Universidad de Salamanca, Instituto de Investigación Biomédica de Salamanca (IBSAL), Campus Miguel de Unamuno s/n, 37007 Salamanca, Spain; 3 Department of Medical Oncology, University Hospital of Salamanca, 37007 Salamanca, Spain; 4 Departamento de Estadistica, Universidad de Salamanca, Instituto de Investigación Biomédica de Salamanca (IBSAL), Campus Miguel de Unamuno s/n, 37007 Salamanca, Spain; 5 Departamento de Derecho del Trabajo y Trabajo Social, Universidad de Salamanca, Campus Miguel de Unamuno s/n, 37007 Salamanca, Spain

**Keywords:** Cancer-related fatigue, Chemotherapy-induced peripheral neuropathy, Colorectal cancer, Resistance training

## Abstract

**Background/objectives:**

Chemotherapy-induced peripheral neuropathy (CIPN) is a frequent toxicity of neurotoxic agents commonly used in colorectal cancer (CRC) and has limited evidence-based management options. Exercise may help, but the optimal prescription during chemotherapy remains uncertain. To evaluate the effects of an eight-week exercise program on CIPN (primary outcome), cancer-related fatigue (CRF), handgrip strength, and physical activity in CRC patients receiving chemotherapy.

**Methods:**

This pilot parallel-group randomized controlled trial screened 44 CRC patients receiving chemotherapy; 40 were randomized (1:1) to an intervention group (supervised resistance training twice weekly plus home-based aerobic exercise) or a control group (home-based physical activity program only). Outcomes were assessed at baseline and after eight weeks. NCT06404359.

**Results:**

Twenty-seven participants completed follow-up (intervention n = 15; control n = 12). Baseline characteristics were comparable. No statistically significant between-group differences were observed for CIPN, CRF, or handgrip strength. Physical activity increased significantly in the intervention group compared with controls (p < 0.001). Adherence exceeded 80%, and no exercise-related adverse events were reported.

**Conclusions:**

In this pilot trial, an eight-week program combining supervised resistance training with home-based aerobic exercise was feasible and safe and increased self-reported physical activity, but did not demonstrate statistically significant improvements in CIPN, fatigue, or strength compared with home-based aerobic exercise alone. Larger adequately powered trials are warranted.

**Supplementary Information:**

The online version contains supplementary material available at https://doi.org/10.1007/s12094-026-04371-z.

## Introduction

Colorectal cancer (CRC) is one of the leading malignant neoplasms worldwide, both due to its high incidence and mortality rates and to the impact it has on the health and quality of life of oncology patients. Globally, CRC is the third most frequently diagnosed cancer and the second leading cause of cancer-related death, surpassed only by lung cancer, accounting for up to 9.3% of all cancer deaths [1]. This burden is particularly relevant in older adults, who often present with greater comorbidity and vulnerability; however, age alone does not consistently determine survival outcomes, as prognosis may also be influenced by stage at diagnosis, tumour location, baseline health status, and treatment received [2]. In recent years, the incidence of CRC has increased, partly due to modifiable risk factors such as obesity and sedentary lifestyles, although evolving diagnostic and classification frameworks may also have influenced some epidemiological trends [3, 4]. Systemic treatment remains a cornerstone of colorectal cancer management and includes chemotherapy, as well as immunotherapy in selected molecular subgroups, particularly tumours with deficient mismatch repair or high microsatellite instability, with the aims of eradicating disease, preventing recurrence, or reducing tumour burden prior to surgery [3, 5].

Although therapeutic advances have contributed to improving survival rates for cancer patients, these same treatments, such as chemotherapy, produce significant side effects that cause toxicity, negatively affecting patients during their treatments [6]. At the same time, advances in biomarker-guided treatment have increased the complexity of CRC management, although treatment-related toxicities remain highly relevant to patients’ function and quality of life [7].

Among the most relevant complications is chemotherapy-induced peripheral neuropathy (CIPN), which is a direct side effect of treatment in cancer patients [8]. CIPN can develop because of treatment with multiple chemotherapeutic agents, including platinum compounds (notably oxaliplatin in CRC), taxanes, alkaloids, thalidomide and bortezomib, more commonly affecting large sensory nerves, leading to paraesthesia, dysaesthesia and numbness in the hands and feet [9]. Acute CIPN may even require dose reduction or treatment interruption, thereby reduce the effectiveness of therapy and increasing overall mortality [10].

Although there are no standard treatment strategies for this condition within the public health system, various studies and investigations highlight the importance of physical exercise in counteracting the progression of CIPN through structured program lasting 12 weeks or longer. During this period, such program improve functional capacity, strength, and balance in oncology patients [11–13].

Other important consequences of CRC and its treatment include cancer related fatigue (CRF), CIPN, decreased levels of physical activity, and loss of muscular strength, all of which can markedly affect patients’ functional independence, adherence to treatment, and overall quality of life. CRF is one of the most prevalent and distressing symptoms in oncology patients [14], characterised by a persistent and subjective sense of physical, emotional, and cognitive tiredness that is disproportionate to recent activity and not relieved by rest. It is closely related to reduced physical performance and motivation, which can, in turn, exacerbate deconditioning and limit participation in rehabilitation program. In parallel, the onset of CIPN leads to sensory and motor deficits, balance disturbances, and gait instability, further restricting mobility and daily functioning [15]. The combination of fatigue and neuropathic symptoms contributes to progressive muscle weakness and reduced strength, particularly in the lower limbs, which increases the risk of falls and functional decline. Together, these symptoms create a vicious cycle that amplifies physical inactivity and compromises both treatment tolerance and recovery in CRC patients [16].

These consequences may be reduced through structured exercise program implemented during treatment, with several studies reporting beneficial effects on physical function, symptoms, and treatment-related side effects [17, 18].

Although longer exercise program can be effective, they may pose a barrier to clinical implementation in CRC patients undergoing chemotherapy, particularly in those in especially vulnerable situations, such as individuals experiencing severe fatigue, neuropathic pain, or treatment-related haematological toxicity. Recent studies have demonstrated that shorter exercise program, typically ranging from 4 to 10 weeks, also lead to significant improvements in strength, balance, and symptoms associated with CIPN. Zimmer et al. [19] implemented an 8 week multimodal program (2 sessions/week, 60 min each) combining endurance, resistance, and balance training in metastatic CRC patients during chemotherapy, resulting in improved balance and strength and stabilization of neuropathic symptoms. Similarly, Singh et al. [20] tested a 10 week supervised aerobic and resistance intervention (2 sessions/week + home aerobic activity) during neoadjuvant chemoradiation in rectal cancer patients, showing enhanced lower-limb strength and functional performance. More recently, Pesce et al. [21] reported that a 4 week multimodal prehabilitation program combining interval and resistance training with home aerobic exercise, nutritional supplementation, and psychological support improved functional capacity before colorectal surgery and maintained gains post-operatively. Collectively, these shorter interventions—ranging from 4 to 10 weeks, with 2–3 supervised sessions per week—demonstrate that structured, multimodal exercise can effectively reduce treatment-related dysfunction, musculoskeletal problems, pain, and other side effects, even when implemented during active treatment or preoperative phases.For these reasons, this study aimed to evaluate the effect of an eight-week structured strength training program combined with a home-based physical activity plan on CIPN, CRF, physical activity levels, and muscle strength loss in CRC patients undergoing active chemotherapy.

## Materials and methods

### Study design, recruitment and registration

A parallel group randomized controlled trial was conducted involving patients undergoing active treatment for CRC, in accordance with CONSORT guidelines [22]. Data collection primarily took place at the Complejo Asistencial Universitario de Salamanca and the Faculty of Nursing and Physiotherapy of the University of Salamanca. Patients were recruited through the Oncology Department of the Complejo Asistencial Universitario de Salamanca and were informed about the study procedure.

The study was approved by the Ethics Committee of the University of Salamanca (approval number 1209) and adhered to the principles outlined in the Declaration of Helsinki. The clinical trial was registered on ClinicalTrials.gov (NCT06404359).

### Participants

All participants received both verbal and written information about the study procedures and provided written informed consent prior to participation. CRC patients were eligible for inclusion if treated with adjuvant chemotherapy or immunotherapy, had no evidence of disease recurrence, were physically inactive (< 60 min of structure exercise/week) in the past eight weeks, had capability to comprehend the assessment procedures and carry out the exercise regimen and were willing to participate in the study voluntarily.

Exclusion criteria were presence of any contraindications for exercise participation (e.g., musculoskeletal disorders, severe cardiovascular conditions, bone metastases, etc.) and patients who could not tolerate the training program prescribed in the study.

### Interventions and procedure

The Oncology Department of the Complejo Asistencial Universitario de Salamanca applied the established eligibility criteria to recruit participants for the study. A total of 40 individuals were enrolled after providing informed consent.

**Fig. 1:**
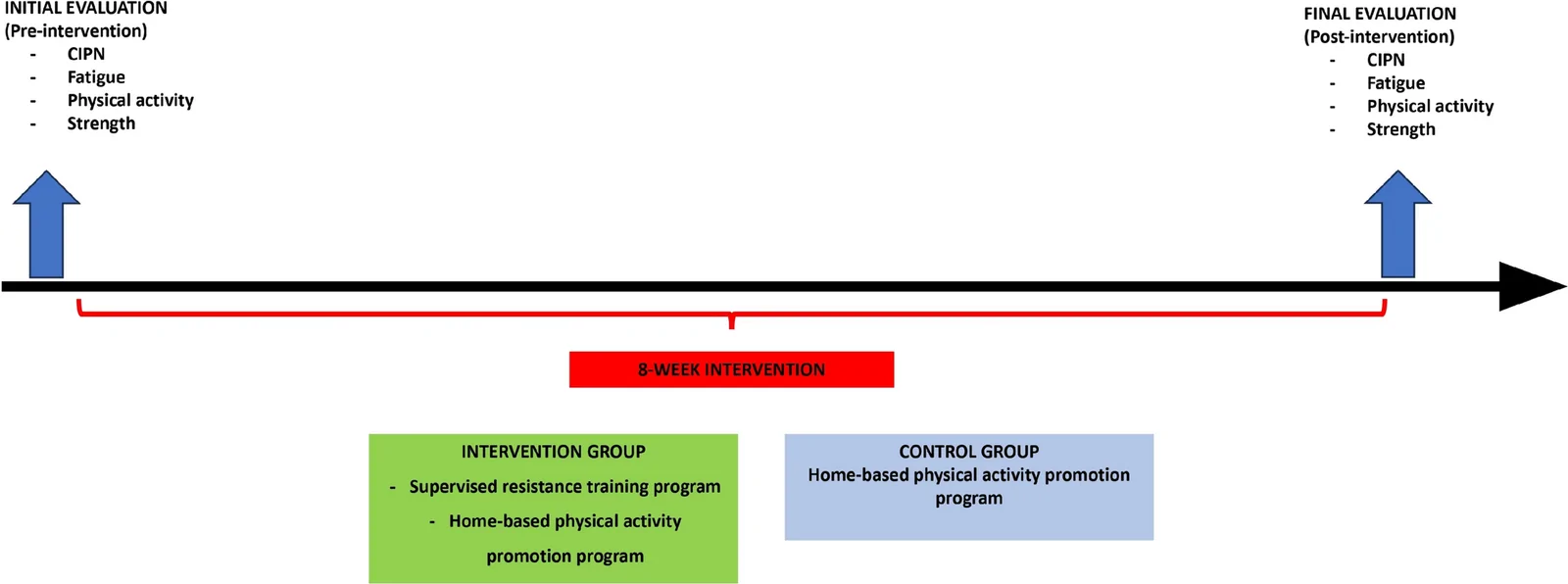
Study design and clinical trial procedure

The physical exercise intervention program was specifically designed for CRC patients undergoing chemotherapy treatment. The training program was focused on resistance exercises, although daily aerobic exercises were also included. Participants were then randomly assigned to either the intervention group (IG, n = 20) or the control group (CG, n = 20). Baseline measurements were obtained from all participants prior to the start of the intervention. The overall study procedure is illustrated in Fig. 1.

The intervention group engaged in a supervised resistance training program twice weekly for a duration of eight weeks, complemented by a home-based physical activity promotion program performed three times per week. All supervised sessions were led by a physiotherapist at a healthcare facility (Supplementary File S1, complete description of the intervention and the home-based physical activity promotion program).

The control group followed a home-based physical activity program three days per week, consisting of three structured exercise sessions per week plus daily walking. To monitor adherence, all participants received weekly phone calls.

#### Exercise prescription (FITT)

*Frequency:* Intervention group—2 supervised resistance training sessions/week plus 3 home-based aerobic sessions/week; Control group—home-based aerobic sessions 5 days/week.

*Intensity:* Resistance training intensity was individualised and progressed based on patient tolerance and symptom fluctuation during chemotherapy; aerobic sessions were prescribed at a moderate intensity (e.g., “talk test”/perceived exertion).

*Time:* Supervised sessions lasted approximately 45–60 min; home-based aerobic sessions were prescribed for at least 20 min per session.

*Type:* Supervised resistance training targeted major muscle groups, and the aerobic component consisted of home-based activities such as brisk walking or cycling. A full session-by-session description is provided in Supplementary File S1.

### Outcomes

All participants were assessed at the baseline and at the end of the study intervention period. In the initial assessment, all variables, including sociodemographic factors, were measured.

#### Chemotherapy-induced peripheral neuropathy

CIPN was evaluated using the European Organisation for the Research and Treatment of Cancer Quality of Life Questionnaire — CIPN20 (EORTC QLQ-CIPN20), Spanish version [23], which includes 20 items divided into three subscales assessing sensory, motor, and autonomic symptoms. Each item was scored on a Likert scale ranging from 1 (“not at all”) to 4 (“very much”). Raw scores were subsequently transformed to a standardized 0–100 scale, with higher scores reflecting greater symptom severity. The Spanish version of the EORTC QLQ-CIPN20 has been culturally adapted and preliminarily validated in oncology populations [24].

#### Cancer-related fatigue

CRF was measured using the 12-item EORTC QLQ-FA12, Spanish [25]. It consists of 12 items, with four response categories for each item, coded with values from 1 to 4. In accordance with the scales of the EORTC QLQ-C30, the FA12 scores are transformed to the range 0–100, with higher levels indicating greater degrees of fatigue. The FA12 comprises three subscales: physical fatigue (five items), emotional fatigue (three items), and cognitive fatigue (two items). The remaining two items serve as global indicators for interference of fatigue with daily activities and social sequelae of fatigue.

#### Physical activity

Overall physical activity was assessed using the Spanish version of the International Physical Activity Questionnaire (IPAQ) [26]. The IPAQ consists of seven items that evaluate the frequency and duration of physical activity across different intensity levels: low activity (< 600 MET minutes/week), such as walking at home, at work, or for leisure; moderate activity (600–3000 MET minutes/week), including activities such as cycling, playing tennis, or carrying light loads; and high activity (> 3000 MET minutes/week), encompassing high-intensity exercises such as aerobic workouts, digging, or heavy lifting. The IPAQ also captures sedentary behavior over the previous week, offering a comprehensive assessment of the participant’s physical activity patterns. The total physical activity score is calculated as the sum of MET-minutes/week from vigorous, moderate, and light activities [27].

#### Strength

Grip strength was measured using the Jamar® dynamometer (J00105, Lafayette Instrument Company, USA), a hydraulic dynamometer widely regarded as the gold standard for assessing grip strength [28]. Both hands will be tested following the Southampton Protocol [29]. The patient was seated in a chair with back support, and the procedure was explained before starting. Three consecutive measurements were taken on each hand, with each contraction lasting 5 s and a 10 s rest interval between measurements. Verbal encouragement was provided during the test to ensure maximum effort. The patient’s positioning included the shoulder adducted and neutrally rotated, elbow flexed at 90°, forearm in a neutral position, and wrist in slight extension (0°–30°). The second Jamar® grip setting was used for all participants, except for those with smaller hands, who used the first setting, with this noted in the report. All measurements were performed by the same evaluator. For the analysis, the mean value obtained from the three attempts for each hand was used.

### Sample size and randomization

The sample size was calculated using G*Power 3.1 statistical software. A two-tailed independent samples t-test was selected for the analysis. The effect size (Cohen’s d = 1.11) was derived from the mean difference in the CIPN scale, as reported in Zimmer et al. [19], due to the similarity of the interventions. Additional reference was made to the study by Bland et al. [30], which employed the same outcome measure (EORTC QLQ-CIPN20) in a similar patient population but with a different intervention. A significance level of 0.05 and a statistical power of 95% were applied. The estimated required sample size was 36 participants. Accounting for an anticipated 20% dropout rate, based on previous findings, the final sample size was adjusted to 44 participants, with 22 allocated to each group.

### Blinding

Given the nature of the study, it was not possible to blind either the participants or the evaluators to the intervention. However, the statistical analysis was performed by an independent statistician who remained unaware of the group assignments.

### Statistical analysis

Quantitative sociodemographic variables are presented as mean ± standard deviation (SD), and qualitative variables as absolute and relative frequencies. The Shapiro–Wilk test was used to assess the normality of the distributions. Baseline between-group comparisons were performed using the independent samples t-test for normally distributed quantitative variables and the chi-square test for qualitative variables.

For variables that did not meet normality assumptions, between-group comparisons were conducted using the Mann–Whitney U test. This approach was used for the variables number of cycles and months of evolution, as well as for pre–post changes in the scales and subscales of the instruments applied. The use of non-parametric analyses was justified by the asymmetrical distributions and the presence of outliers.

For the CIPN, CRF, handgrip strength, and total METs outcomes, medians and their 95% confidence intervals were calculated for each intervention group [31]. Pre–post change scores were used as indicators of change for each scale or subscale, and the difference in medians between the intervention and control groups was used as an estimate of the magnitude of change. Rank-biserial correlation derived from the Mann–Whitney U test [32] was calculated as a standardized effect size, allowing comparison across outcomes. Effect sizes were interpreted as low (< 0.125), moderate (0.125–0.465), or large (> 0.465) [33]. Pre- and post-intervention values are presented in Supplementary File S2.

For IPAQ categories (low, moderate, high), the McNemar test was used to assess within-group changes from pre- to post-intervention. In addition, logistic analysis including the interaction term group × time was performed to examine whether changes in IPAQ category differed between intervention groups. The kappa coefficient was calculated as an effect size for agreement between pre- and post-intervention IPAQ categories.

Statistical significance was set at α = 0.05, and 95% confidence intervals were calculated where appropriate. All analyses were performed using jamovi software, version 2.6 [34].

## Results

**Fig. 2:**
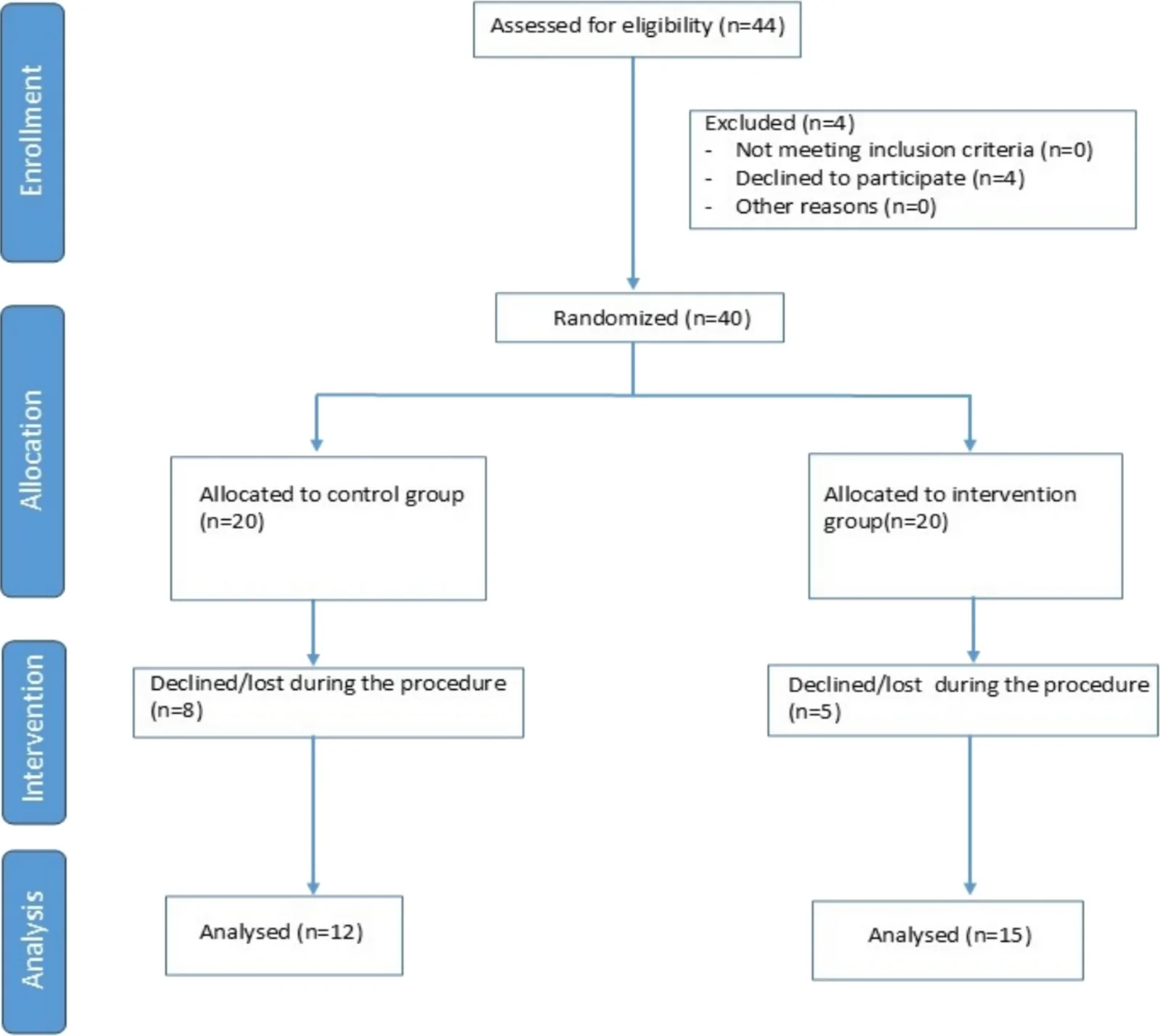
CONSORT flow diagram of participant recruitment, allocation, follow-up and analysis

A total of 44 patients were screened for eligibility; 4 declined participation, and 40 were randomized (20 per group). During the intervention, 13 participants discontinued (5 from the IG and 8 from the CG), primarily due to medical treatment-related adverse effects, scheduling difficulties, or loss of motivation. Consequently, data from 27 participants were analyzed (15 in the IG and 12 in the CG). Details of recruitment and assessment are shown in Fig. 2.

### Sociodemographic information

The demographic characteristics of the IG and the CG are summarized in Table 1. The mean age was comparable between the IG (59.6 ± 8.7 years) and the CG (61.6 ± 13.6 years), with no statistically significant difference (*p* = 0.674). Similarly, the mean BMI was 25.46 ± 4.39 kg/m^2^ in the IG and 24.48 ± 4.28 kg/m^2^ in the CG, with no significant difference observed (*p* = 0.568). No statistically significant differences were detected in the proportion of men and women between the experimental groups (*p*=0.381).

**Table 1:** Sociodemographic and clinical characteristics

Variable	Intervention group (n = 15)	Control group (n = 12)	p-value
Gender, n (%)			
Male	10 (66%)	6 (50%)	0.381
Female	5 (34%)	6 (50%)	
Age (years), mean ± SD	59.6 ± 8.70	61.6 ± 13.61	0.674
Weight (kg), mean ± SD	70.4 ± 12.81	67.5 ± 11.61	0.548
Height (cm), mean ± SD	165.93 ± 7.21	165.92 ± 11.09	0.996
BMI (kg/m^2^), mean ± SD	25.46 ± 4.39	24.48 ± 4.28	0.568
Cancer stage, n (%)			0.241
II	2 (13%)	1 (8%)	
III	6 (40%)	7 (58%)	
IV	7 (47%)	4 (34%)	
Number of cycles, median ± IQR	4 ± 5.50	4.5 ± 7.25	0.657
Type of treatment, n (%)			
QT	8 (53%)	9 (75%)	0.657
IO	1 (6%)	0 (0%)	
QT + IO	6 (41%)	3 (25%)	
Months of evolution, median ± IQR	6 ± 28.5	5.5 ± 5	0.250

*SD* standard deviation, *kg* kilograms, *cm* centimetres, *IQR* interquartile range

### Changes in chemotherapy-induced peripheral neuropathy

Table 2 shows the changes observed in the different dimensions of CIPN. No statistically significant differences were found between groups in any of the evaluated domains. There is a positive trend toward improvement in the sensory subscale in both groups, more pronounced in the intervention group.

**Table 2:** Changes in chemotherapy-induced peripheral neuropathy

Variable	Median difference (95% CI)	p-value		Effect size
Sensory	− 9.26 (− 29.26 to 10.74)	0.464		− 0.17
Motor	14.58 (1.39 to 27.78)	0.360		0.21
Autonomic	0 (–)	0.440		− 0.13

*CI* confidence interval

For the sensory dimension, the median difference was − 9.26 (95% CI − 29.26 to 10.74; *p* = 0.464), with a small effect size (0.17). The motor dimension showed a median difference of 14.58 (95% CI 1.39 to 27.78; *p* = 0.360), also corresponding to a small effect size (0.21). Regarding the autonomic dimension, no relevant changes were detected (median difference = 0; *p* = 0.440; effect size = − 0.13).

Overall, although a slight trend toward improvement in the motor domain was observed, these variations did not reach statistical significance. It should also be noted that in the case of motor domain, the confidence interval indicates significance. This is due to the distribution of observations and the presence of outliers (Supplementary File S3).

### Changes in cancer related fatigue

Table 3 summarizes the changes in the different dimensions of CRF. No statistically significant differences were observed between groups across any of the assessed domains. In the physical dimension of fatigue, a decrease was observed in both groups after the exercise intervention. In the dimension of interference with activities of daily living, a trend toward improvement was noted in the intervention group.

**Table 3:** Changes in CRF

Variable	Median difference (95% CI)	p-value		Effect size
Physical	− 3.33 (− 18.9 to 12.28)	0.258		− 0.26
Emotional	0 (− 11.60 to 11.60)	0.070		− 0.40
Cognitive	0 (–)	0.849		− 0.04
Interference with daily life	− 33.33 (− 56.46 to − 11.20)	0.237		− 0.26
Social sequelae	0 (–)	0.412		− 0.07

*CI* confidence interval

For the physical dimension, the median difference was − 3.33 (95% CI − 18.9 to 12.28; p = 0.258), with a small effect size (− 0.26). The emotional dimension showed a median difference of 0 (95% CI − 11.60 to 11.60; p = 0.07), with a small-to-moderate effect size (− 0.40), suggesting a potential trend toward improvement. The cognitive dimension remained unchanged (median difference = 0; p = 0.849; effect size = − 0.04).

Regarding interference with daily life, a reduction of − 33.33 (95% CI − 56.46 to − 11.20; p = 0.237) was observed, corresponding to a small effect size (− 0.26). Finally, no relevant changes were detected in social sequelae (median difference = 0; p = 0.412; effect size = − 0.07).

Overall, although small improvements were observed in emotional fatigue and interference with daily life, none of these reached statistical significance. As in the case of the motor subscale in the previous section, both the emotional fatigue and interference with daily life subscales show confidence intervals for the medians that contradict the significance test. Therefore, the distribution of observations must be taken into account for a better interpretation of the results (Supplementary File S4A-B).

### Changes in strength

Table 4 presents the changes in handgrip strength for both dominant and non-dominant limbs. No statistically significant differences were observed between groups. Regarding muscle strength, in both the dominant and non-dominant hand, there is a trend toward improvement in the intervention group, more evident in the non-dominant hand.

**Table 4:** Changes in strength

Variable	Median difference (95% CI)	p-value		Effect size
Dominant	2.50 (− 2.55 to 7.55)	0.294		0.24
Non dominant	1.83 (− 3.26 to 6.92)	0.251		0.27

*CI* confidence interval

For the dominant hand, the median difference was 2.50 (95% CI − 2.55 to 7.55; p = 0.294), corresponding to a small effect size (0.24). Similarly, the non-dominant hand showed a median difference of 1.83 (95% CI − 3.26 to 6.92; p = 0.251), with a small effect size (0.27).

Overall, a slight trend toward increased strength was observed in both limbs, although these changes did not reach statistical significance.

### Changes in physical activity

In the pre-post intervention change in the total METs score based on the IPAQ questionnaire, significant differences were detected between the intervention and control groups (*p-value* < *0.001*, median difference = 2226 and 95% CI 1002 to 2451). In contrast, the IPAQ subscales did not show statistically significant differences in the change in category from baseline to post-intervention between the intervention groups.

Table 5 shows the change that occurred in each subscale of Physical activity for the two treatments. The McNemar test did not show significant results for any of the subscales in the different interventions except in the intervention group for IPAQ high. On this subscale, 9 individuals from the intervention group out of the 14 individuals who were not physically active at the pre-test stage became physically active at the post-test stage. This trend is also evident on the IPAQ MODERATE subscale, although less pronounced and not significant, but not on the IPAQ LOW subscale. In any case, these results should be interpreted with caution due to the small sample size.

**Table 5:** Changes in physical activity

Intervention				IPAQ LOW POST		Total
				NO	YES	
IG	IPAQ LOW PRE p-value = -; k = -	NO	Count (%)	11 (100.0)	0.0 (0.0)	11
		YES	Count (%)	4 (100.0)	0.0 (0.0)	4
CG	IPAQ LOW PRE p-value = 0.25-; k = 0.44	NO	Count (%)	7 (100.0)	0.0 (0.0)	7
		YES	Count (%)	3 (60,0)	2 (40,0)	5

				IPAQ MODERATE POST		Total
				NO	YES	
IG	IPAQ MODERATE PRE p-value = 0.219-; k = −0.13	NO	Count (%)	9 (64.3)	5 (35.7)	14
		YES	Count (%)	1 (100.0)	0 (0.0)	1
CG	IPAQ MODERATE PRE p-value = 1.000-; k = 0.31	NO	Count (%)	10 (66.7)	5 (33.3)	15
		YES	Count (%)	5 (71.4)	2 (28.6)	7

				IPAQ HIGH POST		Total
				NO	YES	
IG	IPAQ HIGH PRE p-value = 0.004-; k = 0.10	NO	Count (%)	5 (35.7)	9 (64.3)	14
		YES	Count (%)	0 (0.0)	1 (100.0)	1
CG	IPAQ HIGH PRE p-value = 1-; k = 0.25	NO	Count (%)	5 (33.3)	10 (66.7)	15
		YES	Count (%)	8 (80.0)	2 (20.0)	10

*p-value from McNemar test and κ is Kappa coefficient

*CG* control group, *IG* intervention group

### Adherence and side effects

Adherence to the training program was very good, exceeding 80% in both groups. No adverse events related to the exercise program were reported in either group during the intervention period.

## Discussion

This study expands on previous evidence regarding the feasibility and safety of implementing an eight week physical exercise program in patients with CRC undergoing chemotherapy. The results show a trend toward improvement in CIPN, muscle strength, and CRF, although most variables did not reach statistical significance. These findings should be interpreted considering the small sample size and the short duration of the intervention (8 weeks), factors that likely limited the magnitude of the effects and their statistical significance.

Starting with the interpretation of the results, a trend toward improvement was observed in the sensory subscale of CIPN in both groups, more noticeable in the intervention group with supervised exercise. Previous studies have shown that 8 to 12 week multimodal exercise programs can reduce symptoms associated with CIPN and improve balance and muscle strength in CRC patients undergoing chemotherapy. Some authors reported significant improvements in CIPN in CRC patients after an eight week combined program [19, 35]. Other previous studies found a decrease in neuropathic symptoms in cancer survivors who completed longer strength and balance programs [11, 36]. Compared to the results obtained in our study, although statistical significance was not achieved, a positive trend was observed in the sensory subscale, which is consistent with the hypothesis that physical exercise may help attenuate this symptom, in line with previous literature. However, these findings should be interpreted with caution, as the observed changes did not reach statistical significance in our sample.

Muscle strength improved in both upper limbs, measured in the dominant and non-dominant hand, with a greater effect size achieved in the non-dominant hand. Previous literature proves the effectiveness of strength training in maintaining and improving muscle strength in patients undergoing active treatment for cancer. One previous study showed that a 10 week combined program in patients with rectal cancer undergoing neoadjuvant therapy significantly improved strength and functional capacity [20]. Most studies’ findings indicated that endurance training had potential benefits over time on muscle strength and endurance, coinciding with the results of our intervention [37–39].

A decrease in CRF was observed in both study groups, mainly focused on the physical dimension, although there was also a noticeable trend toward improvement in interference with activities of daily living in the supervised exercise intervention group. The scientific evidence available to date attributes a fundamental role to physical exercise as a weapon in the fight against fatigue related to cancer or its treatments. Improvements in this variable are more evident in long-term exercise programs, lasting more than 12 weeks, and in those that use combined strength training and aerobic exercise training plans [40–42].

In these last two variables analyzed, muscle strength and CRF, the lack of statistical significance in some areas observed in our study may be mainly due to the short duration of the intervention. Improvements in these areas are more evident in training programs lasting longer than 12 weeks, with progressive load and professional supervision. Training periods longer than 8 weeks are likely to be necessary to achieve clinically relevant benefits in muscle strength and CRF, as well as analyzing larger study samples to reduce variability among participants.

In terms of total physical activity levels recorded, a significant increase was observed after completing both exercise programs, with metabolic expenditure increasing in both groups. This indicates that the intervention promoted the adoption of healthy lifestyle habits, in addition to achieving improvements in functional parameters. In this regard, the findings are consistent with the existing literature, which shows that exercise programs carried out during active cancer treatments, such as chemotherapy, improve treatment adherence and have a significant effect on maintaining previous levels of physical activity, as well as on acquiring healthier lifestyle habits. This improves patients’ quality of life and ensures greater therapeutic continuity [12].

Finally, the applied physical exercise program combining strength training and aerobic training was safe and easily applicable in this population, consistent with existing literature [20, 41].

Overall, the results of this study suggest that a strength training program combined with daily aerobic exercise is feasible and safe in CRC patients undergoing chemotherapy and may contribute to improvements in CIPN, muscle strength, and CRF. However, as most of these changes did not reach statistical significance, the findings should be considered preliminary. Similarly, the participants’ level of physical activity increased, promoting the adoption of healthier lifestyles. However, the improvements observed in some of the variables did not reach statistical significance, probably due to the small sample size that limited statistical power, the clinical heterogeneity of the participants (different cancer stages and treatment plans), and the short duration of the training programs; possibly 8 weeks were insufficient to achieve more robust physiological adaptations.

### Implications of the study

This study supports the feasibility of incorporating exercise programs into the care of patients undergoing chemotherapy, even in short-term formats that combine supervised sessions with home-based activity. The findings suggest that the training program evaluated was feasible, well tolerated, and may help promote active lifestyles in patients with CRC. Nevertheless, larger adequately powered studies are needed to determine its effectiveness on CIPN, CRF, and muscle strength.

### Limitations

The results of this study should be analyzed considering its limitations. First, the sample size was small and there were losses of participants during follow-up, which reduced the possibility of detecting statistically significant differences. Furthermore, the 8 week duration of the program may not have been sufficient to consolidate the benefits obtained in CIPN, muscle strength, and CRF, although it did promote adherence to training, which was always above 80%. The absence of blinding is another methodological limitation of the study. In addition, the heterogeneity of systemic treatment exposure should be acknowledged, as participants received chemotherapy, immunotherapy, or combined regimens, which may have influenced symptom burden and responsiveness to exercise.

To increase knowledge in these areas, future studies should consider larger samples, extend the intervention period to at least 12 weeks, and perform subgroup analyses according to cancer stage and type of treatment. It is also of interest to explore hybrid approaches that combine supervised and home-based components. Long-term follow-up would be valuable to assess whether initial improvements are maintained over time and whether they translate into greater adherence to cancer treatment and a better quality of life.

## Conclusions

In conclusion, this pilot study suggests that an 8 week physical exercise program focused on strength training combined with aerobic activity is feasible, safe, and well tolerated in patients with CRC undergoing active chemotherapy. The intervention was associated with an increase in self-reported physical activity, whereas no statistically significant between-group differences were observed for CIPN, CRF, or handgrip strength. Although some variables showed favorable trends, these findings should be interpreted cautiously given the small sample size and short intervention period. Larger and longer clinical trials are warranted to clarify the effectiveness of this approach.

## Supplementary Information

Below is the link to the electronic supplementary material.

Supplementary file1 (DOCX 172 KB)

## Data Availability

The data presented in this study are available on request from the corresponding author. The data are not publicly available due to privacy and ethical restrictions.

## Abbreviations

CRC: Colorectal cancer
CIPN: Chemotherapy-induced peripheral neuropathy
CRF: Cancer related fatigue
IG: Intervention group
CG: Control group
EORTC QLQ-CIPN20: European Organisation for the Research and Treatment of Cancer Quality of Life CIPN 20 Questionnaire
EORTC QLQ-FA12: European Organisation for the Research and Treatment of Cancer Quality of Life Fatigue Questionnaire
IPAQ: Spanish Version of the International Physical Activity Questionnaire

## References

[1] Bray F, Laversanne M, Sung H, Ferlay J, Siegel RL, Soerjomataram I, et al. Global cancer statistics 2022: GLOBOCAN estimates of incidence and mortality worldwide for 36 cancers in 185 countries. CA Cancer J Clin. 2024;74:229–63. 10.3322/caac.21834. 38572751

[2] Osseis M, Nehmeh WA, Rassy N, Derienne J, Noun R, Salloum C, et al. Surgery for T4 colorectal cancer in older patients: determinants of outcomes. JPM. 2022;12:1534. 10.3390/jpm12091534. PMC9504737 36143319

[3] An S, Park S. Association of physical activity and sedentary behavior with the risk of colorectal cancer. J Korean Med Sci. 2022;37:e158. 10.3346/jkms.2022.37.e158. PMC9110266 35578589

[4] Mathew BG, Aliyuda F, Taiwo D, Adekeye K, Agada G, Sanchez E, et al. From biology to diagnosis and treatment: the ariadne’s thread in cancer of unknown primary. IJMS. 2023;24:5588. 10.3390/ijms24065588. PMC10053301 36982662

[5] Adeleke S, Haslam A, Choy A, Diaz-Cano S, Galante JR, Mikropoulos C, et al. Microsatellite instability testing in colorectal patients with lynch syndrome: lessons learned from a case report and how to avoid such pitfalls. Per Med. 2022;19:277–86. 10.2217/pme-2021-0128. 35708161

[6] Katta B, Vijayakumar C, Dutta S, Dubashi B, Nelamangala Ramakrishnaiah VP. The incidence and severity of patient-reported side effects of chemotherapy in routine clinical care: a prospective observational study. Cureus [Internet]. 2023 [cited 2025 Nov 12]. 10.7759/cureus.38301 PMC10226821 37261144

[7] Kyrochristou I, Lianos GD, Kyrochristou GD, Anagnostopoulos G, Bali C, Boussios S, et al. Agnostic biomarkers and molecular signatures in colorectal cancer-guiding chemotherapy and predicting response. Biomed. 2025;13:2038. 10.3390/biomedicines13082038. PMC12383588 40868288

[8] Loprinzi CL, Lacchetti C, Bleeker J, Cavaletti G, Chauhan C, Hertz DL, et al. Prevention and management of chemotherapy-induced peripheral neuropathy in survivors of adult cancers: ASCO guideline update. JCO. 2020;38:3325–48. 10.1200/JCO.20.01399. 32663120

[9] Park SB, Goldstein D, Krishnan AV, Lin CS, Friedlander ML, Cassidy J, et al. Chemotherapy-induced peripheral neurotoxicity: a critical analysis. CA Cancer J Clin. 2013;63:419–37. 10.3322/caac.21204. 24590861

[10] Seretny M, Currie GL, Sena ES, Ramnarine S, Grant R, MacLeod MR, et al. Incidence, prevalence, and predictors of chemotherapy-induced peripheral neuropathy: a systematic review and meta-analysis. Pain. 2014;155:2461–70. 10.1016/j.pain.2014.09.020. 25261162

[11] Kneis S, Wehrle A, Müller J, Maurer C, Ihorst G, Gollhofer A, et al. It’s never too late - balance and endurance training improves functional performance, quality of life, and alleviates neuropathic symptoms in cancer survivors suffering from chemotherapy-induced peripheral neuropathy: results of a randomized controlled trial. BMC Cancer. 2019;19:414. 10.1186/s12885-019-5522-7. PMC6498676 31046719

[12] Hatlevoll I, Oldervoll LM, Wibe A, Stene GB, Stafne SN, Hofsli E. Physical exercise during adjuvant chemotherapy for colorectal cancer—a non-randomized feasibility study. Support Care Cancer. 2021;29:2993–3008. 10.1007/s00520-020-05789-z. PMC8062327 33030598

[13] Streckmann F, Balke M, Cavaletti G, Toscanelli A, Bloch W, Décard BF, et al. Exercise and neuropathy: systematic review with meta-analysis. Sports Med. 2022;52:1043–65. 10.1007/s40279-021-01596-6. 34964950

[14] Amarelo A, Da Mota MCC, Amarelo BLP, Ferreira MC, Fernandes CS. Effects of physical exercise on chemotherapy-induced peripheral neuropathy: a systematic review and meta-analysis. Pain Manag Nurs. 2025;26:212–21. 10.1016/j.pmn.2024.12.011. 39855997

[15] Stoller S, Capozza S, Alberti P, Lustberg M, Kleckner IR. Framework to leverage physical therapists for the assessment and treatment of chemotherapy-induced peripheral neurotoxicity (CIPN). Support Care Cancer. 2023;31:293. 10.1007/s00520-023-07734-2. PMC11552664 37086308

[16] Haas S, Mikkelsen AH, Kronborg CJS, Oggesen BT, Møller PF, Fassov J, et al. Management of treatment-related sequelae following colorectal cancer. Colorectal Dis. 2023;25:458–88. 10.1111/codi.16299. 35969031

[17] Siebert S, Kersten J, Tomanek A, Heinz S, Niels T, Baumann FT. Lending a hand: supportive exercise therapy for cancer treatment-induced polyneuropathy of the upper extremity—VISCIPH A. Support Care Cancer. 2025;33:712. 10.1007/s00520-025-09712-2. PMC12283904 40694173

[18] Soriano-Maldonado A, Díez-Fernández DM, Esteban-Simón A, Rodríguez-Pérez MA, Artés-Rodríguez E, Casimiro-Artés MA, et al. Effects of a 12 week supervised resistance training program, combined with home-based physical activity, on physical fitness and quality of life in female breast cancer survivors: the EFICAN randomized controlled trial. J Cancer Surviv. 2023;17:1371–85. 10.1007/s11764-022-01192-1. PMC10442259 35314958

[19] Zimmer P, Trebing S, Timmers-Trebing U, Schenk A, Paust R, Bloch W, et al. Eight week, multimodal exercise counteracts a progress of chemotherapy-induced peripheral neuropathy and improves balance and strength in metastasized colorectal cancer patients: a randomized controlled trial. Support Care Cancer. 2018;26:615–24. 10.1007/s00520-017-3875-5. 28963591

[20] Singh F, Galvão DA, Newton RU, Spry NA, Baker MK, Taaffe DR. Feasibility and preliminary efficacy of a 10 week resistance and aerobic exercise intervention during neoadjuvant Chemoradiation treatment in rectal cancer patients. Integr Cancer Ther. 2018;17:952–9. 10.1177/1534735418781736. PMC6142076 29888608

[21] Pesce A, Fabbri N, Colombari S, Uccellatori L, Grazzi G, Lordi R, et al. A randomized controlled clinical trial on multimodal prehabilitation in colorectal cancer patients to improve functional capacity: preliminary results. Surg Endosc. 2024;38:7440–50. 10.1007/s00464-024-11198-8. PMC11614948 39210058

[22] Moher D, Hopewell S, Schulz KF, Montori V, Gotzsche PC, Devereaux PJ, et al. CONSORT 2010 Explanation and elaboration: updated guidelines for reporting parallel group randomised trials. BMJ. 2010;340:c869–c869. 10.1136/bmj.c869. PMC2844943 20332511

[23] Roser Velasco Fargas, T. J. Postma, Neil Aaronson, M. Simo, Jordi Bruna. Preliminary validation of the eortc chemotherapy-induced peripheral neuropathy quality of life questionnaire (qlq-cipn20) spanish version in a series of multiple myeloma patients treated with bortezomib. Neuro-oncology. Brusells; 2010.

[24] Postma TJ, Aaronson NK, Heimans JJ, Muller MJ, Hildebrand JG, Delattre JY, et al. The development of an EORTC quality of life questionnaire to assess chemotherapy-induced peripheral neuropathy: the QLQ-CIPN20. Eur J Cancer. 2005;41:1135–9. 10.1016/j.ejca.2005.02.012. 15911236

[25] Weis J, Tomaszewski KA, Hammerlid E, Ignacio Arraras J, Conroy T, Lanceley A, et al. International psychometric validation of an EORTC quality of life module measuring cancer related fatigue (EORTC QLQ-FA12). JNCI: J Natl Cancer Inst [Internet]. 2017 [cited 2025 Sep 1]. 109. 10.1093/jnci/djw273 28376231

[26] Craig CL, Marshall AL, Michael M, Bauman AE, Booth ML, Ainsworth BE, et al. International physical activity questionnaire: 12-Country reliability and validity. Med Sci Sports Exerc. 2003;35:1381–95. 10.1249/01.MSS.0000078924.61453.FB. 12900694

[27] Hurtig-Wennlöf A, Hagströmer M, Olsson LA. The International physical activity questionnaire modified for the elderly: aspects of validity and feasibility. Public Health Nutr. 2010;13:1847–54. 10.1017/S1368980010000157. 20196910

[28] Roberts HC, Denison HJ, Martin HJ, Patel HP, Syddall H, Cooper C, et al. A review of the measurement of grip strength in clinical and epidemiological studies: towards a standardised approach. Age Ageing. 2011;40:423–9. 10.1093/ageing/afr051. 21624928

[29] Dodds RM, Syddall HE, Cooper R, Kuh D, Cooper C, Sayer AA. Global variation in grip strength: a systematic review and meta-analysis of normative data. Age Ageing. 2016;45:209–16. 10.1093/ageing/afv192. PMC4776623 26790455

[30] Bland KA, Kirkham AA, Bovard J, Shenkier T, Zucker D, McKenzie DC, et al. Effect of exercise on Taxane chemotherapy-induced peripheral neuropathy in women with breast cancer: a randomized controlled trial. Clin Breast Cancer. 2019;19:411–22. 10.1016/j.clbc.2019.05.013. 31601479

[31] Bonett DG, Price RM. Statistical inference for a linear function of medians: confidence intervals, hypothesis testing, and sample size requirements. Psychol Methods. 2002;7:370–83. 10.1037/1082-989X.7.3.370. 12243307

[32] Bakan D. The test of significance in psychological research. Psychol Bull. 1966;66:423–37. 10.1037/h0020412. 5974619

[33] Cohen J. Statistical Power Analysis for the Behavioral Sciences [Internet]. 0 ed. Routledge. 2013 [cited 2025 Mar 16]. 10.4324/9780203771587

[34] The jamovi project [Internet]. 2025. https://www.jamovi.org

[35] Schönsteiner SS, Bauder Mißbach H, Benner A, Mack S, Hamel T, Orth M, et al. A randomized exploratory phase 2 study in patients with chemotherapy-related peripheral neuropathy evaluating whole-body vibration training as adjunct to an integrated program including massage, passive mobilization and physical exercises. Exp Hematol Oncol. 2017;6:5. 10.1186/s40164-017-0065-6. PMC5297221 28194306

[36] Guo S, Han W, Wang P, Wang X, Fang X. Effects of exercise on chemotherapy-induced peripheral neuropathy in cancer patients: a systematic review and meta-analysis. J Cancer Surviv. 2023;17:318–31. 10.1007/s11764-022-01182-3. 35149899

[37] Ligibel JA, Bohlke K, May AM, Clinton SK, Demark-Wahnefried W, Gilchrist SC, et al. Exercise, diet, and weight management during cancer treatment: ASCO guideline. JCO Am Soc Clin Oncol (ASCO). 2022;40:2491–507. 10.1200/jco.22.00687. 35576506

[38] Van Rooijen SJ, Engelen MA, Scheede-Bergdahl C, Carli F, Roumen RMH, Slooter GD, et al. Systematic review of exercise training in colorectal cancer patients during treatment. Scand Med Sci Sports. 2018;28:360–70. 10.1111/sms.12907. 28488799

[39] Chen S-C, Huang H-P, Huang W-S, Lin Y-C, Chu T-P, Beaton RD, et al. Non-randomized preliminary study of an education and elastic-band resistance exercise program on severity of neuropathy, physical function, muscle strength and endurance and quality of life in colorectal cancer patients experiencing oxaliplatin-induced peripheral neuropathy. Eur J Oncol Nurs. 2020;49:101834. 10.1016/j.ejon.2020.101834. 33120223

[40] Lu Y, Qu H-Q, Chen F-Y, Li X-T, Cai L, Chen S, et al. Effect of Baduanjin Qigong exercise on cancer-related fatigue in patients with colorectal cancer undergoing chemotherapy: a randomized controlled trial. Oncol Res Treat. 2019;42:431–9. 10.1159/000501127. PMC6771138 31266043

[41] Demmelmaier I, Brooke HL, Henriksson A, Mazzoni A, Bjørke ACH, Igelström H, et al. Does exercise intensity matter for fatigue during (neo-)adjuvant cancer treatment? The phys-can randomized clinical trial. Scand Med Sci Sports. 2021;31:1144–59. 10.1111/sms.13930. 33527488

[42] Poort H, Peters MEWJ, Van Der Graaf WTA, Nieuwkerk PT, Van De Wouw AJ, van Der Nijhuis- Sanden MWG, et al. Cognitive behavioral therapy or graded exercise therapy compared with usual care for severe fatigue in patients with advanced cancer during treatment: a randomized controlled trial. Ann Oncol. 2020;31(1):115–22. 10.1016/j.annonc.2019.09.002. 31912784

