# Supplementary material for: Effects of a resistance training program on chemotherapy-induced peripheral neuropathy, cancer-related fatigue, muscle strength and physical activity in colorectal cancer patients undergoing chemotherapy: a pilot randomized controlled trial
Source: Clin Transl Oncol. 2026 Apr 30;28(10):4794–805. doi: 10.1007/s12094-026-04371-z (PMC13601180; doi:10.1007/s12094-026-04371-z)
Supplement: Supplementary file 1 [file 12094_2026_4371_MOESM1_ESM.docx]

# Supplementary File S1: Supervised resistance training programme

## SESSION 1

1.- Warm-up (10 minutes)

- 2 minutes: Walk in circles taking advantage of the width of the room incorporating mobility exercises.
- 20 seconds: Just walk.
- 20 seconds: Shoulder circles forward.
- 20 seconds: Shoulder circles backward.
- 20 seconds: Alternating knee lifts to chest.
- 20 seconds: Walk on tiptoes.
- 20 seconds: Hip separation walking.
- 2 minutes: Single-leg balance with slight knee and hip flexion, 1 minute on each leg.
- 1 minute: Wall/floor push-ups.
- 1 minute: Wall sit.
- 2 minutes: Skipping.
- 2 minutes: Ball toss in pairs or against a wall.

2.- Resistance Training

- Normal squat.
- Romanian deadlift.
- Abdominal plank (30 seconds).
- Bench press with barbell.
- Bicep curls with dumbbells.
- Dumbbell rows.

**There will be 6 stations, and participants will rotate from one to the next until completing all 6 exercises. They will do 3 sets of 12 repetitions at 70% of 1RM. Rest 30 seconds between stations and at the end of each complete set, rest for 90 seconds.*

3.- Cool-down

- 3 minutes: Slow-paced walking with diaphragmatic breathing.
- 1 minute: Quadriceps stretch.
- 1 minute: Hamstring stretch.
- 1 minute: Calf stretch.
- 1 minute: Bicep stretch.
- 1 minute: Shoulder stretch.
- 2 minutes: Seated cervical mobility exercises.

## SESSION 2

1.- Warm-up (10 minutes)

- 2 minutes: Walk in circles taking advantage of the room's width incorporating mobility exercises.
- 20 seconds: Just walk.
- 20 seconds: Shoulder abduction/adduction in transverse plane.
- 20 seconds: Alternating shoulder flexion/extension.
- 20 seconds: Leg lifts with straight knees alternately.
- 20 seconds: Heel walks.
- 20 seconds: Jump in place.
- 2 minutes: Ankle proprioception on single-leg support, 1 minute on each leg.
- 1 minute: Knee lift with contralateral arm raise.
- 1 minute: Bodyweight squats/sit and stand from a chair.
- 2 minutes: Step-up with contralateral knee raise.
- 2 minutes: Boxing (punching forward with alternate arms at a slow pace).

2.- Resistance Training

- Lunge.
- Glute bridge with barbell/kettlebell.
- Abdominal crunches.
- Dumbbell bench press.
- Shoulder press.
- Sit-to-stand jumps.

**There will be 6 stations, and participants will rotate from one to the next until completing all 6 exercises. They will do 3 sets of 12 repetitions at 70% of 1RM. Rest 30 seconds between stations and at the end of each complete set, rest for 90 seconds*

3.- Cool down

- 3 minutes: Slow-paced walking with diaphragmatic breathing.
- 1 minute: Quadriceps stretch.
- 1 minute: Hamstring stretch.
- 1 minute: Calf stretch.
- 1 minute: Bicep stretch.
- 1 minute: Shoulder stretch.
- 2 minutes: Seated cervical mobility exercises.

## SESSION 3

1.- Warm-up (10 minutes)

- 2 minutes: Walk in circles taking advantage of the room's width incorporating mobility exercises.
- 20 seconds: Just walk.
- 20 seconds: Elbow flexion/extension.
- 20 seconds: Shoulder abduction/adduction.
- 20 seconds: Hip flexion + abduction.
- 20 seconds: Walking lunges.
- 20 seconds: Side steps.
- 2 minutes: Lateral step-ups, 1 minute on each leg.
- 1 minute: Close-grip push-ups on wall/floor.
- 1 minute: Bodyweight alternating lunges in place.
- 2 minutes: Jumping jacks.
- 2 minutes: Bodyweight glute bridges.

2.- Resistance Training

- Wide stance squat.
- Deadlift.
- Barbell rows.
- Barbell bicep curls.
- Dumbbell lateral raises.
- Single-leg box step-ups.

**There will be 6 stations, and participants will rotate from one to the next until completing all 6 exercises. They will do 3 sets of 12 repetitions at 70% of 1RM. Rest 30 seconds between stations and at the end of each complete set, rest for 90 seconds.*

3.- Cool-down

- 3 minutes: Slow-paced walking with diaphragmatic breathing.
- 1 minute: Quadriceps stretch.
- 1 minute: Glute stretch.
- 1 minute: Hamstring stretch.
- 1 minute: Bicep stretch.
- 1 minute: Shoulder stretch.
- 2 minutes: Seated cervical mobility exercises.

## SESSION 4

1.- Warm-up (10 minutes)

- 2 minutes: Walk in circles taking advantage of the room's width incorporating mobility exercises.
- 20 seconds: Just walk.
- 20 seconds: Squat and stand up as you walk.
- 20 seconds: Trunk rotations with shoulder push.
- 20 seconds: Shoulder raises.
- 20 seconds: Side lunges.
- 20 seconds: Walk backward.
- 2 minutes: Sit and stand from a chair.
- 1 minute: Theraband elbow flexion.
- 1 minute: Calf raises and holds statically.
- 2 minutes: Jumping in place.
- 2 minutes: Shoulder circumduction with light weight.

2.- Resistance Training

- Lateral lunge.
- Deadlift.
- Incline bench chest fly with dumbbells.
- Supine tricep press.
- Front shoulder raises with plate.
- Jump squats.

**There will be 6 stations, and participants will rotate from one to the next until completing all 6 exercises. They will do 3 sets of 12 repetitions at 70% of 1RM. Rest 30 seconds between stations and at the end of each complete set, rest for 90 seconds.*

3.- Cool-down

- 3 minutes: Slow-paced walking with diaphragmatic breathing.
- 1 minute: Quadriceps stretch.
- 1 minute: Glute stretch.
- 1 minute: Tricep stretch.
- 1 minute: Chest stretch.
- 1 minute: Shoulder stretch.
- 2 minutes: Seated cervical mobility exercises.

# HOME-BASED PHYSICAL ACTIVITY PLAN

The following exercises should be performed three days per week (each day you will do the five exercises assigned to day one, day two, or day three). These sessions should not coincide with the days of physiotherapist-led training.

Each exercise should be performed for 1 minute, followed by 1 minute of rest before starting the next exercise. You should complete 2 full sets of the 5 exercises. In total, the daily programme will take around 20 minutes.

Additionally, you must walk for 1 hour every day. DAY 1:

- Sit-to-stand from a chair.
- Lateral lunge.
- Supine cycling (lying on your back).
- Elbow flexion.
- Lateral shoulder raise.

DAY 2:

- Lunge.
- Quadriceps wall sit (isometric hold).
- Glute bridge (lying on your back).
- Frontal shoulder raise.
- Wall push-ups. DAY 3:
- Tiptoe walking.
- Squats.
- Knee-to-chest raise.
- Abdominal crunches.
- Shoulder press.

*Example: On Tuesday, perform the five exercises from DAY 1; on Wednesday, the five exercises from DAY 2; and on Friday, the five exercises from DAY 3. You must walk for 1 hour every day.

**Supplementary File S2: PRE-POST values**

| *Chemotherapy-induced peripheral neuropathy (PRE-POST values)* | | | | | | |
| --- | --- | --- | --- | --- | --- | --- |
|  | **Group** | **n** | **Mean** | **Median** | **SD** | **IQR** |
| *Sensory* | *Intervention (PRE)* | 15 | 24.44 | 22.22 | 21.08 | 16.67 |
|  | *Control (PRE)* | 12 | 29.32 | 25.93 | 20.13 | 33.33 |
|  | *Intervention (POST)* | 15 | 9.44 | 4.17 | 10.96 | 18.75 |
|  | *Control (POST)* | 12 | 21.18 | 27.08 | 13.93 | 22.92 |
| *Motor* | *Intervention (PRE)* | 15 | 11.11 | 4.17 | 15.00 | 12.50 |
|  | *Control (PRE)* | 12 | 16.67 | 16.67 | 14.10 | 26.04 |
|  | *Intervention (POST)* | 15 | 4.44 | 0.00 | 10.96 | 2.08 |
|  | *Control (POST)* | 12 | 3.47 | 4.17 | 2.99 | 4.17 |
| *Autonomic* | *Intervention (PRE)* | 15 | 2.96 | 0.00 | 6.60 | 0.00 |
|  | *Control (PRE)* | 12 | 3.70 | 0.00 | 7.24 | 2.78 |
|  | *Intervention (POST)* | 15 | 2.59 | 0.00 | 5.50 | 0.00 |
|  | *Control (POST)* | 12 | 6.94 | 0.00 | 15.19 | 2.78 |

 SD = Standard Deviation; IQR= Interquartile Range

| *Cancer Related Fatigue (PRE-POST values)* | | | | | | | |
| --- | --- | --- | --- | --- | --- | --- | --- |
|  | | **Group** | **n** | **Mean** | **Median** | **SD** | **IQR** |
| *Physical* | *Intervention (PRE)* | | 15 | 21.78 | 13.33 | 20.07 | 23.33 |
|  | *Control (PRE)* | | 12 | 18.89 | 16.67 | 13.88 | 15.00 |
|  | *Intervention (POST)* | | 15 | 6.22 | 6.67 | 7.33 | 10.00 |
|  | *Control (POST)* | | 12 | 13.89 | 10.00 | 12.54 | 16.67 |
| *Emotional* | *Intervention (PRE)* | | 15 | 12.59 | 11.11 | 13.84 | 22.22 |
|  | *Control (PRE)* | | 12 | 6.48 | 0.00 | 8.81 | 11.11 |
|  | *Intervention (POST)* | | 15 | 2.96 | 0.00 | 6.60 | 0.00 |
|  | *Control (POST)* | | 12 | 9.26 | 5.56 | 11.44 | 13.89 |
| *Cognitive* | *Intervention (PRE)* | | 15 | 4.44 | 0.00 | 9.89 | 0.00 |
|  | *Control (PRE)* | | 12 | 4.17 | 0.00 | 10.36 | 0.00 |
|  | *Intervention (POST)* | | 15 | 1.11 | 0.00 | 4.30 | 0.00 |
|  | *Control (POST)* | | 12 | 1.39 | 0.00 | 4.81 | 0.00 |
| *Social*  *Sequelae* | *Intervention (PRE)* | | 15 | 4.44 | 0.00 | 17.21 | 0.00 |
|  | *Control (PRE)* | | 12 | 0.00 | 0.00 | 0.00 | 0.00 |
|  | *Intervention (POST)* | | 15 | 0.00 | 0 | 0.00 | 0.00 |
|  | *Control (POST)* | | 12 | 0.00 | 0.00 | 0.00 | 0.00 |
| *Interference with daily life* | *Intervention (PRE)* | | 15 | 28.89 | 33.33 | 27.79 | 50.00 |
|  | *Control (PRE)* | | 12 | 27.78 | 33.33 | 19.25 | 8.33 |
|  | *Intervention (POST)* | | 15 | 4.44 | 0.00 | 11.73 | 0.00 |
|  | *Control (POST)* | | 12 | 13.89 | 0.00 | 22.29 | 33.33 |

 SD = Standard Deviation; IQR= Interquartile Range

| *Strength (PRE-POST values)* | | | | | | |
| --- | --- | --- | --- | --- | --- | --- |
|  | *Group* | **n** | **Mean** | **Median** | **SD** | **IQR** |
| *Dominant* | *Intervention (PRE)* | 15 | 24.3 | 20.3 | 7.81 | 10.67 |
|  | *Control (PRE)* | 12 | 14.6 | 17.5 | 7.55 | 10.25 |
|  | *Intervention (POST)* | 15 | 24.3 | 23.0 | 10.13 | 10.17 |
|  | *Control (POST)* | 12 | 13.2 | 13.3 | 8.39 | 9.08 |
| *No Dominant* | *Intervention (PRE)* | 15 | 22.3 | 19.3 | 8.34 | 7.17 |
|  | *Control (PRE)* | 12 | 13.1 | 13.3 | 5.71 | 6.58 |
|  | *Intervention (POST)* | 15 | 24.3 | 21.0 | 8.02 | 9.33 |
|  | *Control (POST)* | 12 | 11.9 | 12.0 | 7.09 | 8.75 |

 SD = Standard Deviation; IQR= Interquartile Range

**Supplementary File S3: Outliers CIPN**

**Supplementary File S4A: Outliers CRF interference with daily life**

**Supplementary File S4B: Outliers CRF emotional**
